# YOLOv8-Seg with Dynamic Multi-Kernel Learning for Infrared Gas Leak Segmentation: A Weakly Supervised Approach

**DOI:** 10.3390/s25164939

**Published:** 2025-08-10

**Authors:** Haoyang Shen, Lushuai Xu, Mingyue Wang, Shaohua Dong, Qingqing Xu, Feng Li, Haiyang Yu

**Affiliations:** 1 College of Carbon Neutral Energy, China University of Petroleum, Beijing 102249, China; haoyangs078@163.com; 2 College of Artificial Intelligence, China University of Petroleum, Beijing 102249, China; 202131101@student.cup.edu.cn; 3 Key Laboratory of Oil and Gas Safety and Emergency Technology, Ministry of Emergency Management, Beijing 102249, China; xuqq@cup.edu.cn (Q.X.); yhy@student.cup.edu.cn (H.Y.); 4 School of Engineering, China University of Petroleum-Beijing at Karamay, Karamay 834000, China; wangmy@cup.edu.cn; 5 College of Safety and Ocean Engineering, China University of Petroleum, Beijing 102249, China; 6 Petrochina Beijing Gas Pipeline Co., Ltd., Beijing 100101, China; lifeng23@pipechina.com.cn

**Keywords:** YOLOv8-seg, infrared gas leak, mask generation, multi-branch collaboration, confidence weighted

## Abstract

Gas leak detection in oil and gas processing facilities is a critical component of the safety production monitoring system. Non-contact detection technology based on infrared imaging has emerged as a vital real-time monitoring method due to its rapid response and extensive coverage. However, existing pixel-level segmentation networks face challenges such as insufficient segmentation accuracy, rough gas edges, and jagged boundaries. To address these issues, this study proposes a novel pixel-level segmentation network training framework based on anchor box annotation and enhances the segmentation performance of the YOLOv8-seg network for gas detection applications. First, a dynamic threshold is introduced using the Visual Background Extractor (ViBe) method, which, in combination with the YOLOv8-det network, generates binary masks to serve as training masks. Next, a segmentation head architecture is designed, incorporating dynamic kernels and multi-branch collaboration. This architecture utilizes feature concatenation under deformable convolution and attention mechanisms to replace parts of the original segmentation head, thereby enhancing the extraction of detailed gas features and reducing dependency on anchor boxes during segmentation. Finally, a joint Dice-BCE (Binary Cross-Entropy) loss, weighted by ViBe-CRF (Conditional Random Fields) confidence, is employed to replace the original Seg_loss. This effectively reduces roughness and jaggedness at gas edges, significantly improving segmentation accuracy. Experimental results indicate that the improved network achieves a 6.4% increase in F1 score and a 7.6% improvement in the mIoU (mean Intersection over Union) metric. This advancement provides a new, real-time, and efficient detection algorithm for infrared imaging of gas leaks in oil and gas processing facilities. Furthermore, it introduces a low-cost weakly supervised learning approach for training pixel-level segmentation networks.

## 1. Introduction

Oil and gas facilities represent critical nodes within the petrochemical supply chain, with their safe operation heavily reliant on the secure transportation and storage of industrial gases such as methane, ethylene, and carbon dioxide. Amid rapid economic growth, the scale of industrial gas utilization has expanded significantly. However, this increase has been paralleled by a surge in gas leak incidents, posing substantial challenges to safety management in the petrochemical industry [1,2,3]. Consequently, the comprehensive and accurate deployment of gas leak detection systems in these facilities has emerged as an urgent priority requiring immediate attention.

In recent years, advancements in infrared thermal imaging technology have established infrared spectroscopy-based gas detection as a vital research domain in fields such as environmental monitoring and petrochemical engineering. Common industrial gases, including methane and ethylene, exhibit distinct absorption bands within the infrared spectrum, making them detectable by specialized infrared thermal imaging equipment [4]. Compared to conventional point-contact sensors, these devices offer notable advantages, including an extended detection range and non-contact measurement capabilities. Furthermore, they visualize gas leaks as images, delivering intuitive and immediate feedback to technical personnel. Despite the high precision of current handheld and stationary infrared detection systems, the complexity of the resulting image data necessitates expert analysis, thereby elevating labor costs. As such, the development of intelligent image segmentation techniques has become a pivotal research focus to enhance the accessibility and widespread adoption of infrared thermal imaging technology [5,6,7,8,9].

Integrating computer vision techniques into the analysis of infrared images is a crucial method for achieving high-precision, low-cost real-time detection. Infrared gases often fail to completely absorb background radiation, causing a portion of the background radiation energy to enter the imaging system, thereby reducing the contrast between the foreground and the background. This results in difficulties in accurately locating gas plume regions [10]. Image denoising and enhancement techniques are vital for improving the accuracy of gas detection. In the field of image denoising, methods such as Gaussian median filters [11], bilateral filters [12], and wavelet transforms [13] are commonly used. Image enhancement techniques improve the contrast between the foreground and background by refining histogram processing methods [14].

Due to the diffusion characteristics of gases, they generally exhibit strong motion properties in space. Consequently, motion detection algorithms, such as frame differencing [15], background subtraction [16], and the ViBe algorithm [17], can be used for detecting such phenomena. However, these methods often suffer from significant accuracy loss in complex environments. Wu et al. [18] applied a cascaded enhancement algorithm to enhance gas regions in infrared images, followed by differential operations and connected domain filtering to eliminate noise. Duan et al. [19] introduced a real-time feature extraction method based on frame differencing to effectively remove “ghosting” artifacts and incorporated an adaptive threshold strategy during foreground judgment to remove residual noise. Although traditional motion detection methods exhibit low computational overhead, they require frequent iterations to effectively capture inter-frame dynamics. In practical industrial scenarios characterized by complex backgrounds, irregular gas dispersion patterns, and interference from moving objects, these methods struggle to distinguish gaseous targets from non-gaseous entities. Consequently, they demonstrate significantly elevated false positive rates, failing to meet actual industrial detection requirements.

With the rapid development of deep learning technologies, significant advances have been made in the field of infrared gas leakage detection [20]. Current research focuses on constructing intelligent detection frameworks based on convolutional neural networks (CNNs) and temporal prediction methods, which involve tasks such as object detection, image contrast enhancement, and gas plume localization. Zhou et al. [21] constructed a multi-scale feature extraction network based on ConvNext and integrated coordinate attention and segmentation attention modules into the YOLO network to enhance detection performance. Huang et al. [22] proposed the GLRNet model, which employs temporal difference inputs and time-shifting operations to emulate human perceptual mechanisms. By integrating physically constrained features with multi-layer convolutional neural networks, the method enhances gas leakage identification accuracy. However, its low frame rate during image processing impedes effective capture of dynamic changes in high-intensity leakage scenarios such as high-pressure pipeline ruptures.

Wang et al. [23] approached methane leakage scale estimation as a video classification task, developing the VideoGasNet architecture based on 3D CNNs to extract features from RGB frame sequences for improved classification accuracy. Nevertheless, this method requires extensive annotated video data—a critical limitation given the scarcity of gas leakage samples in real industrial settings and prohibitive annotation costs, resulting in constrained model generalizability. While these advanced methods enable automated leakage detection and gas region localization, they exhibit significant limitations in achieving precise gas plume boundary segmentation during extreme leakage events at oil/gas stations—particularly in challenging scenarios including small-volume gas leaks and high-wind-speed dispersion conditions. This segmentation deficiency precludes accurate extraction of critical quantitative parameters, consequently failing to provide evidence-based support for emergency response decision-making in time-sensitive scenarios.

Semantic segmentation, which assigns semantic class labels to each pixel in an image for pixel-level scene construction, has been widely applied across various fields [24,25,26]. The earliest fully convolutional network (FCN) [27] achieved feature learning and spatial prediction integration through an end-to-end encoder-decoder architecture. Subsequently, U-Net [28] introduced skip connections to enable multi-scale feature fusion, while the Deeplab series [29] incorporated dilated convolutions and spatial pyramid pooling to optimize object boundary localization. In recent years, an increasing number of researchers have combined deep learning methods with semantic segmentation models for gas detection. Wang et al. [30] used MobileNetV2 as a backbone network and incorporated the DenseASPP module to enhance the original DeeplabV3+ semantic segmentation model. However, this approach exhibits high inference latency during image processing, while its lightweight backbone variants demonstrate elevated missed-detection rates for low-concentration leaks. Wu et al. [31] introduced an optimized HRNet architecture incorporating hybrid dilated convolution modules and multi-level data-dependent feature aggregation modules, enhancing recognition capability for multi-scale irregular objects. Nevertheless, these methods still require substantial improvements in segmentation accuracy—particularly at plume boundaries—along with real-time performance and edge detail reconstruction fidelity. Furthermore, their heavy reliance on pixel-level annotated data creates dual constraints of poor generalization in extreme leakage scenarios and prohibitive annotation costs, ultimately compromising industrial deployment viability.

To address the aforementioned issues, this study employs the YOLOv8-seg model, known for its rapid inference speed, to handle pixel-level segmentation of infrared gas leakage. Additionally, optimization strategies for both the network architecture and mask annotation workload are proposed. By introducing the dynamic threshold ViBe method in combination with the YOLOv8-det model, a semantic segmentation mask generation algorithm based on anchor box annotations is presented, significantly reducing the manual annotation workload. To tackle issues of anchor box truncation and discontinuous segmentation when processing gases with diverse morphologies using the YOLOv8-seg model, a dynamic kernel segmentation head structure is designed. This structure integrates deformable convolutions and a multi-head attention mechanism, replacing the original segmentation head architecture. To address the jagged edge problem in the results of the semantic segmentation model, an adaptive ViBe-CRF confidence-weighted BCE-Dice joint loss function is proposed, further optimizing the segmentation performance.

The main contributions of this study are summarized as follows:

(1) A ViBe-YOLO collaborative mask generation mechanism, introducing adaptive thresholds and object detection algorithms to construct a weakly supervised learning framework, providing an anchor box annotation solution for gas pixel-level seg-mentation.
(2) A hybrid supervised loss function combining mask generation and Conditional Random Fields, effectively addressing the jagged edges of the foreground in pix-el-level segmentation, optimizing the confidence propagation process, and miti-gating interference from unreliable labels.

A segmentation head structure integrating dynamic kernels and deformable convolutions, solving the high dependency on anchor boxes in the original YOLOv8-seg model. Given the irregular diffusion characteristics of gases, a multi-kernel prediction branch is designed to learn gas characteristics across different dimensions. Finally, an attention mechanism module is introduced to select feature concatenation and restore the true physical properties of the gas.

## 2. Infrared Gas Imaging Data Collection and Mask Generation

### 2.1. Construction of a Realistic Infrared Gas Imaging Dataset

The experimental setup is based on a gas leakage simulation platform for real oil and gas processing facilities, which integrates a flow control module, pressure regulation system, uncertain environmental simulation device, and safety monitoring system. High-purity methane (99% CH_4_, 1% H_2_) was selected as the target gas. Although methane is colorless and odorless, it exhibits a distinct absorption peak in the infrared wavelength of 3.3 μm. Therefore, an in-house developed, domestically produced, cooled industrial infrared thermal imager, GL1000i (Guangzhou Keyi Optoelectronic Technology Co., Ltd., Guangzhou, Guangdong, China), was used for imaging. The device parameters are provided in Table 1.

**Figure 1:**
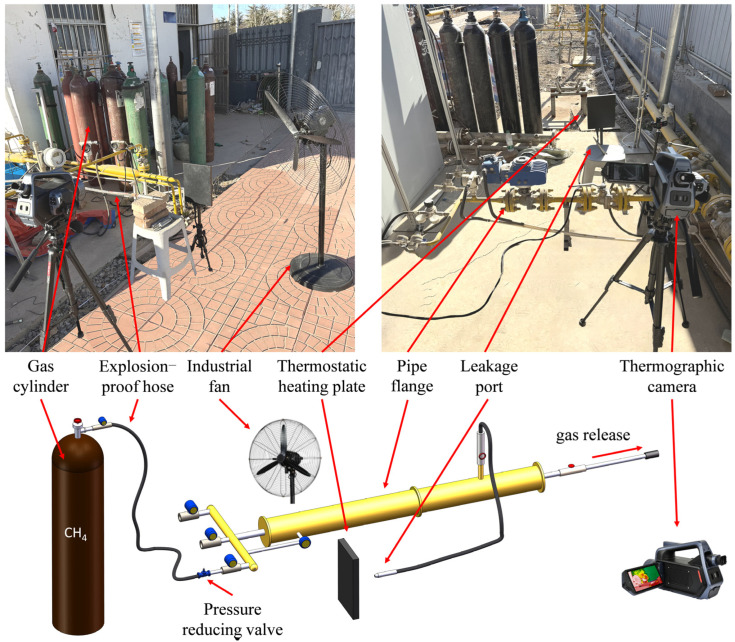
Experimental setup for dataset collection.

Various leakage methods were employed during the experiment. The leakage port was connected to the pipe flange via an explosion-proof hose. The pressure gradient between the gas cylinder and the pipeline flange was controlled using a three-stage pressure-reducing valve. A mass flow meter (ACU10FA-LM, Beijing ACCUFLOW Technology Co., Ltd., Beijing, Beijing, China) was installed at the valve interface to precisely measure the leakage flow rate. Additionally, a thermostatic heating plate and industrial fan were installed in the leakage vent area to regulate the ambient temperature and airflow during the experiment. A methane concentration alarm system (MX2100) was deployed in the experimental area to ensure safety throughout the process. The experimental setup is shown in Figure 1.

Based on the aforementioned experimental platform, a variety of experimental conditions were designed, including shooting distances (5 m, 10 m, 15 m), leakage aperture sizes (1 mm, 2 mm, 3 mm, 4 mm), pipeline gas pressure (ranging from 0.05 MPa to 2 MPa), and environmental wind speeds (1 m/s, 3 m/s, 4 m/s, 5 m/s). Moreover, the experiment considered different leakage port occlusions and complex background scenarios to enhance the diversity of the dataset. During the experiment, the infrared thermal imager continuously captured images under stable conditions with a frame rate of 30 frames per second. A total of 102 video clips were collected, each ranging from 20 to 60 s in duration. After processing with equidistant frame subtraction, a total of 35,120 images were obtained, of which 11,770 images were used for the test set and 23,350 images for the training set.

### 2.2. Mask Annotation Generation Algorithm Integrating Adaptive ViBe and YOLOv8-Det

An innovative method for obtaining segmentation annotations of leaking gas plumes involves using the adaptive ViBe [32] (Visual Background Extractor) and YOLO-det model [33] to process infrared datasets, generating gas region masks that match infrared image sequences in real-world scenarios. The core idea is to first use ViBe to generate preliminary gas region candidates, then leverage the object detection capability of the YOLOv8-det model to filter out noise—replacing traditional morphological post-processing steps. This approach eliminates the enormous workload cost of manual annotation with Labelme software (5.6.1) while maintaining high annotation accuracy.

To reduce the complexity of features learned by YOLOv8-det and improve its prediction accuracy, the infrared image sequence is preprocessed using the ViBe method after frame differencing. This method involves background modeling through random sampling of each pixel’s neighborhood and is particularly well-suited to adapting to dynamic background changes. In practical image processing tasks under real operating conditions, the non-leakage scenario is used to initialize the background samples of the image sequence. A dynamic threshold R is introduced to adaptively update pixel features for different frames, as shown in Equation (1). By comparing each pixel value to the background sample set, pixels are marked as background if a certain number of matching samples meet the criteria. The introduction of the dynamic threshold *R* allows real-time adjustment of the matching standard to accommodate unexpected situations, such as object motion and shadow interference in the background.

(1) $$R=\left\{\begin{array}{ll} R_{0}(1-\varphi ) & \mathrm{if}\;A_{\mathrm{mix}}>\gamma ,\\ R_{0}(1+\varphi ) & \mathrm{else}, \end{array}\right.$$

(2) $$A_{\mathrm{mix}}=\beta_{1}\left|C_{\mathrm{cur}}-C_{\mathrm{prev}}\right|+\beta_{2}\left|d_{\mathrm{cur}}-d_{\mathrm{prev}}\right|.$$

where *R*_0_ is the initial pixel value, *φ* is the adjustment amplitude (through experimental validation, *φ* is optimally set to 0.1 in this study), *C* and *d* represent the mean pixel grayscale and pixel standard deviation of the current frame (cur) and the previous frame (prev), *β* is the weight coefficient, and *γ* is the change determination threshold (through experimental validation, and *γ* is optimally set to 0.5 in this study).

Although the binarized masks output by ViBe can initially separate gas from the background, they often contain dynamic noise and discrete false detection points. Therefore, the semantic discrimination capability of the YOLOv8-det model is utilized to achieve noise reduction. In the YOLOv8-det network, the enhanced feature pyramid (FPN) and path aggregation network (PAN) provide top-down and bottom-up feature flows, ensuring effective detection of targets at different scales (large and small) for gas leaks.

**Figure 2:**
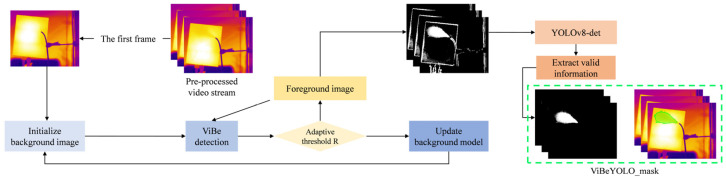
Diagram of mask generation using adaptive ViBe and YOLOv8-det.

This paper uses the YOLOv8n-det network as the main object detection network model. Anchor boxes are annotated on the binarized image frames generated by the adaptive ViBe algorithm using LabelImg software(1.8.6), which are input into the YOLOv8-det network as the training dataset. This preprocessing step effectively converts RGB infrared color images into binarized images, thereby enhancing the dominant features of gas distribution (as shown in Figure 2). During the data annotation phase, a total of 1152 images were manually annotated. The sample size was expanded to 5048 images through data augmentation strategies, including directional flipping (rotating each image in four directions: 0°, 90°, 180°, and 270°) and randomly selecting some long-distance captured samples (experimental groups at 10 m and 15 m) for magnification processing. The ratio of the test set, training set, and validation set is 1:3:1. The final output from the network retains only the gas regions with confidence scores greater than 0.7, which are then used as mask data for subsequent optimization of the YOLOv8-seg network. The masks generated by this method for subsequent training are indicated by the green regions in the original infrared image shown in Figure 2.

In real-world gas leakage scenarios, critical environmental factors such as wind speed, temperature, leakage source characteristics, and release mechanisms must be rigorously considered. Current pixel-level segmentation models typically demand extensive manual annotation, incurring substantial time and financial costs. While conventional approaches like the Gaussian Mixture Model (GMM) exhibit lower computational complexity for gas region separation, their precision remains critically insufficient. To address this limitation, current mainstream methodologies rely on procedurally generated gas plumes through computer software, which are subsequently superimposed onto real infrared backgrounds to create synthetic datasets. However, this approach is often only suitable for open, simple-background environments and is inadequate for complex real-world settings like oil and gas processing facilities, where it fails to provide accurate or effective simulations and lacks a close alignment with actual physical phenomena. Therefore, this paper proposes an innovative method by transferring anchor box annotation techniques from object detection to pixel-level segmentation tasks, significantly reducing the manual annotation workload and costs. Furthermore, it demonstrates that pixel-level segmentation networks, when built upon existing object detection algorithms, can achieve unsupervised learning.

## 3. Methodology

**Figure 3:**
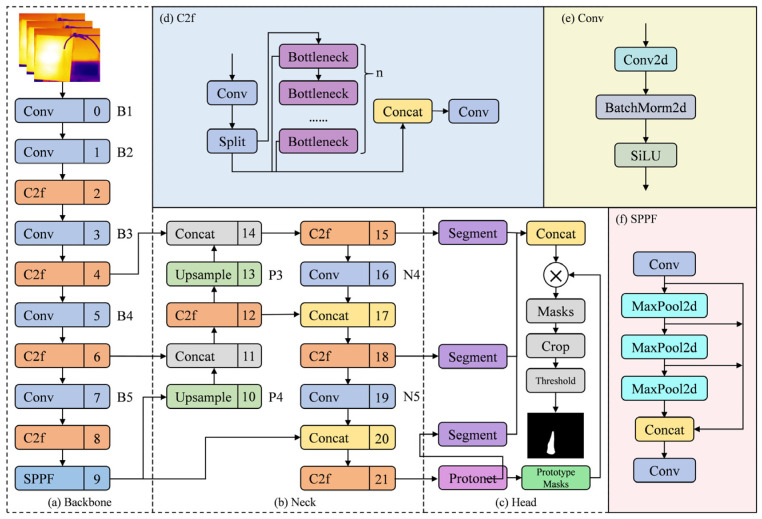
Schematic diagram of the YOLOv8-seg network structure. (**a**) Backbone; (**b**) Neck; (**c**) Head; (**d**) C2f; (**e**) Conv; (**f**) SPPF.

Aiming at the demand for real-time and accurate detection of gas leakage in oil and gas stations, this paper improves the YOLOv8-seg model. The network structure is shown in Figure 3, which is mainly composed of three parts: Backbone, Neck, and Head. In the Backbone, the C2f module is used to replace the C3 module in previous versions. The Neck part adopts the FPN+PAN structure, which realizes the efficient aggregation of multi-scale features through bidirectional feature flow, can take into account gas targets of different sizes, and effectively improve the segmentation accuracy. The Head part adopts a decoupled head structure, abandons the traditional anchor point mechanism, and converts it into anchor-free detection, which can effectively reduce the number of box predictions and accelerate the non-maximum suppression process.

The mask generation mechanism of the YOLOv8-seg network first generates a globally shared prototype mask based on the Protonet network of the P3 layer. The coefficients of the mask are output by the segment mask heads of the P3, P4, and P5 layers, and the prototype masks are weighted and combined through the weight coefficients to generate specific masks for target prediction.

In this paper, dynamic kernels are added to the segmentation head of the YOLOv8-seg network, deformable convolutions are introduced to increase the spatial modeling ability, and finally an attention mechanism is fused for multi-kernel collaborative segmentation of gas regions. In terms of loss function, a spatial prior-guided confidence refinement module (CRF) is introduced, and a Dice-BCE multi-criterion optimization objective function is combined.

### 3.1. Design of Adaptive Dynamic Kernels and Multi-Branch Collaborative Segmentation Head

**Figure 4:**
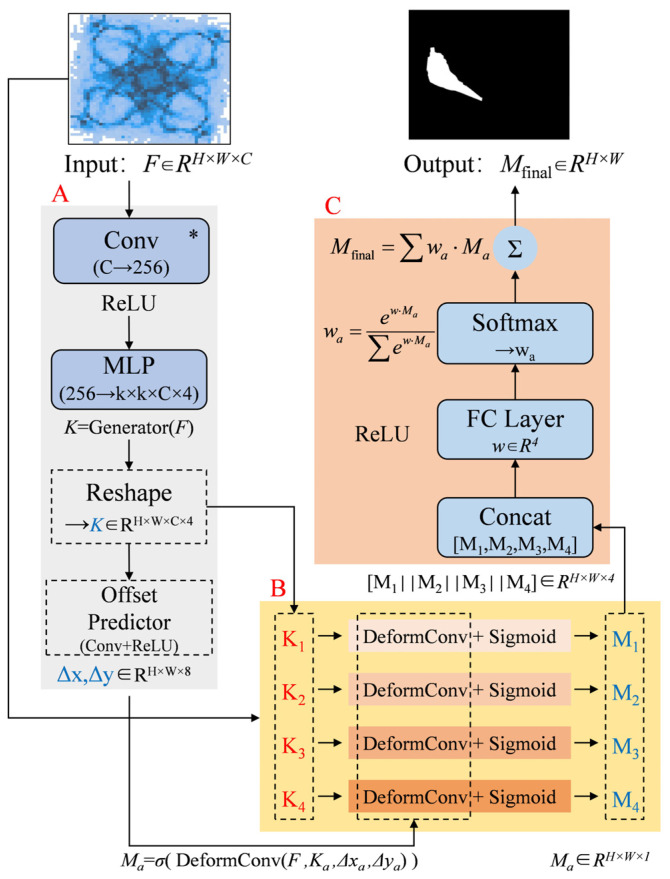
Segmentation head structure with multi-branch dynamic kernels and attention mechanism. (**A**) Dynamic kernel generation; (**B**) Multi-kernel collaboration; (**C**) Attention fusion.

The segmentation head of the YOLOv8-seg model uses a decoupled head design, which generates mask coefficients in addition to predicting bounding boxes and class labels to support instance segmentation [33]. Given the often-variable shape of leaked gases, dynamic kernel generation modules, multi-core collaborative prediction branches, and attention fusion modules are introduced to enhance the segmentation accuracy and generalization ability of the model. The improved segmentation head structure is shown in Figure 4.

Unlike the fixed convolution kernels in the original network, lightweight dynamic convolution kernels are designed to adapt their size and shape according to the feature map. This adaptation addresses the issue of varying gas leak shapes in real-world detection. Specifically, the design consists of two subunits: the kernel parameter generator and the offset predictor. The kernel parameter generator utilizes a 1 × 1 convolution and MLP to form a lightweight network that outputs N dynamic kernels (N is optimally set to 4 herein, following systematic experimental optimization), while the offset predictor introduces a prediction of spatial offset fields to enhance the local deformation capability during the sampling process.

Building on the multi-scale feature extraction for different-sized target features, a multi-branch collaborative mechanism is proposed. Four independent convolution kernels focus on distinct regions: the high-temperature center of the gas diffusion, the diffusion edge, horizontal diffusion, and vertical diffusion patterns, preventing interference between different gas morphologies. Each dynamic kernel branch performs spatially adaptive convolution operations on the feature map, as described in Equation (3).

(3) $$M_{a}=\sigma \left(\mathrm{DeformConv}\left(F,K_{a},\Delta x_{a},\Delta y_{a}\right)\right)$$

where *σ* is the Sigmoid activation function, *F* is the input feature map, and *K_a_* represents the dynamic kernels; (Δ*x_a_*, Δ*y_a_*) are the spatial offset fields.

The attention-weighted fusion module optimizes the multi-branch collaborative process by dynamically adjusting the output weights of different branches. The concatenation process is designed as shown in Equation (4).

(4) $$w=\mathrm{Softmax}\left(W^{T}\left[M_{1}||M_{2}||M_{3}||M_{4}\right]\right)$$

(5) $$M_{\mathrm{final}}=\sum_{i=1}^{4}w_{i}\cdot M_{i}$$

where *w* is the weight vector, *W* ∈ *R*^4 × 4^ is the learnable parameter matrix, || represents the channel concatenation operation, and *M*_1_ to *M*_4_ are the mask predictions from each branch, with *M*_final_ being the final output mask image.

### 3.2. ViBe-CRF Confidence-Weighted Dice-BCE Hybrid Loss

The YOLOv8-seg model includes four primary loss components: box_loss, cls_loss, dfl_loss, and seg_loss. Considering that the Mask samples generated by ViBe and YOLOv8 are used as training data, this study adjusts the weights of Box_loss and dfl_loss and focuses on modifying seg_loss using ViBe-CRF confidence weighting to improve the accuracy of the generated masks. The overall loss function is defined as follows in Equation (6).

(6) $$L=\lambda_{\mathrm{box}}L_{\mathrm{box}}+\lambda_{\mathrm{seg}}L_{\mathrm{seg}}+\lambda_{\mathrm{cls}}L_{\mathrm{cls}}+\lambda_{\mathrm{dff}}L_{\mathrm{dfl}}$$

where *λ* is a hyperparameter used to balance the various loss terms, and *L* represents the individual loss components.

To improve edge segmentation accuracy in gas leakage regions, this study designs the seg_loss to combine pixel-level accuracy of BCE loss with the stability of Dice loss under class imbalance, as described in Equation (7). The BCE loss for each pixel (h,w) is calculated as the cross-entropy between the predicted and true probability, defined in Equation (8). The Dice loss is defined as the ratio of the intersection over the union (IoU) between the predicted and true masks, as shown in Equation (9), and is used to address the class imbalance issue in the dataset.

(7) $$L_{\mathrm{seg}}=\frac{1}{N_{\mathrm{pos}}}\sum_{i=1}^{N_{\mathrm{pos}}}\left[W_{i}\cdot L_{\mathrm{BCE}}\left(p_{i},g_{i}\right)+\lambda_{\mathrm{dice}}W_{i}\cdot L_{\mathrm{Dice}}\left(p_{i},g_{i}\right)\right]$$

(8) $$L_{\mathrm{BCE}}=-\frac{1}{H\cdot W}\sum_{h=1}^{H}\sum_{w=1}^{W}\left[g_{h,w}\log p_{h,w}+\left(1-g_{h,w}\right)\log\left(1-p_{h,w}\right)\right]$$

(9) $$L_{\mathrm{Dice}}=1-\frac{2\sum_{h,w}p_{h,w}g_{h,w}+\epsilon }{\sum_{h,w}p_{h,w}+\sum_{h,w}g_{h,w}+\epsilon }$$

where *N*_pos_ is the number of positive samples (samples with high IoU with the true region), *p* is the predicted mask probability for the positive samples, and *g* is the true mask. *W_i_* represents the dynamic sample weights, and *λ*_dice_ is the weight coefficient for Dice loss. *H* and *W* are the height and width of the mask, while *ϵ* is set to 1 × 10^−5^ to avoid division by zero errors.

The dynamic sample weighting mechanism in Equation (9) combines the motion detection confidence generated by the ViBe-YOLO algorithm with the confidence obtained from the CRF post-processing. This serves as a spatially guided correction module to suppress unreliable pseudo-labels. The dynamic weight is defined in Equation (10).

(10) $$W_{i}=\alpha \cdot C_{\mathrm{vibe}}(i)+(1-\alpha )\cdot C_{\mathrm{crf}}(i)$$

where *α* is the balancing coefficient (*α* is optimally set to 0.7 herein, following systematic experimental optimization) and *C* represents the confidence level. This approach allows the sample weights to be adaptively adjusted during training based on foreground motion features and spatial consistency, thereby enhancing the model’s robustness against difficult-to-segment samples.

## 4. Experiments and Analysis

### 4.1. Model Training

All training in this study was conducted on a Windows 11 operating system using Python 3.8.5, with the PyTorch (2.7.1+cu118) framework, and accelerated by CUDA version 8.0. The hardware environment includes an Intel Core i7-10875H CPU (Intel Corporation, Santa Clara, CA, USA), 16 GB of memory, an NVIDIA GeForce RTX 3070 Ti Laptop GPU, and 8192 MiB of GPU memory. The AdamW optimizer was used during training, with different decay strategies applied to the model’s weight groups. All experiments were conducted using 5-fold cross-validation, with each fold trained for 100 epochs and a batch size of eight to ensure statistical robustness.

### 4.2. Evaluation Metrics

To comprehensively evaluate the performance of the pixel-level segmentation model on the infrared gas leakage dataset, several evaluation metrics were employed, including IoU, F1 score, Dice Similarity Coefficient (DSC), Latency, and GFLOPs [34]. The gas region is defined as the positive sample, while the non-gas region is considered the negative sample, as shown in Table 2.

In the evaluation metrics, precision is used to measure the proportion of true positive instances among all instances predicted as positive by the model, while recall measures the proportion of true positive instances that are correctly predicted as positive. The F1 score comprehensively considers both the precision and recall of the model. The calculation methods for these three metrics are shown in Equations (11)–(13). IoU and DSC measure the overlap between the predicted and true regions, while GFLOPs are used to assess the computational complexity and efficiency of the model.

(11) $$\mathrm{Pr}=\frac{\mathrm{TP}}{\mathrm{TP}+\mathrm{FP}}$$

(12) Re=TPTP+FP

(13) F1=2×Pr×RePr+Re

where Pr represents precision; Re represents recall; TP represents true positives; FP represents false positives; and FN represents false negatives.

### 4.3. Comparison of Loss Function Performance

This study introduces the confidence-weighted CRF-based Dice-BCE loss to replace the original Seg_loss of the network and adjusts the weights of Box_loss and dfl_loss. To validate its effectiveness, comparative experiments were conducted under the same conditions on the following configurations: baseline (YOLOv8-seg), adding only Dice loss, adding only dynamic weights, and the proposed loss function. To accurately reflect the learning effects of the model with different loss functions, the evaluation metrics were computed based on the masks generated by the ViBe and YOLOv8-det models, which provided favorable results. Moreover, the dynamic kernel multi-branch structure and attention mechanism were integrated into the segmentation head for all experimental groups. The experimental results are shown in Table 3.

As shown in Table 3, the loss function designed in this study achieved F1 score and mIoU scores of 73.8% and 67.4%, respectively, outperforming the baseline YOLOv8-seg method by 6.4 and 7.6 percentage points in these two metrics. Compared to the other loss function configurations, it still maintained the best performance, proving the advantage of introducing ViBe and CRF-based confidence for segmenting gas boundary regions. Furthermore, the GFLOPs only increased by approximately 1% compared to the baseline, and the inference latency increased by just 0.2 ms, indicating that the proposed method significantly improves segmentation performance with only a slight increase in computational load, thus demonstrating the practical applicability of the function.

### 4.4. Performance Comparison with Other Algorithms

To further verify the segmentation performance of the improved network on infrared gas leakage image frames in practical scenarios, this study selected several mainstream semantic segmentation models for comparison, including SegFormer [35], Swin-Transformer [36], DeeplabV3+, and the original YOLOv8-seg network. All comparative models were trained under the same training conditions, specifically including the same training dataset, data augmentation strategies, optimizer parameters (learning rate, batch size, etc.), training epochs, and hardware environment. The performance of each network was evaluated using the F1 score and mean IoU (mIoU) as metrics.

Table 4 presents the segmentation results of each model. The experimental results show that the modified network structure outperforms the mainstream semantic segmentation models in both F1 score and IoU metrics, achieving improvements of up to 73.8% and 67.4%, respectively. Compared to the original network, the F1 score improved by 6.4 percentage points, and mIoU increased by 7.6 percentage points. This demonstrates that the proposed improvements provide higher accuracy and segmentation performance for the gas leakage instance segmentation task and can effectively address the real-time detection of gas leaks.

Furthermore, in terms of computational complexity and suitability for real-time applications, experimental results indicate that the improved network achieves an inference speed of 20.1 ms. The inference time per image varies by no more than 3% compared to the original network, and it significantly outperforms other mainstream segmentation networks, which demonstrates the capability to process gas leakage information in oil and gas stations in real time.

To further assess the method’s stability, we first conducted 5-fold cross-validation. The improved YOLOv8-seg network achieved a mIoU of 67.4% ± 1.7%, outperforming the baseline model (59.8% ± 2.5%) and demonstrating enhanced robustness across data partitions. To establish statistical significance, both models underwent ten independent training runs under identical configurations. A two-tailed t-test confirmed the 7.6% mIoU improvement was significant (t = 7.94, *p* = 0.00015), rejecting random variability. The improved model’s narrower 95% confidence interval (66.2–68.6% vs. baseline: 58.2–61.5%) further validates its stability.

### 4.5. Visualization Analysis

**Figure 5:**
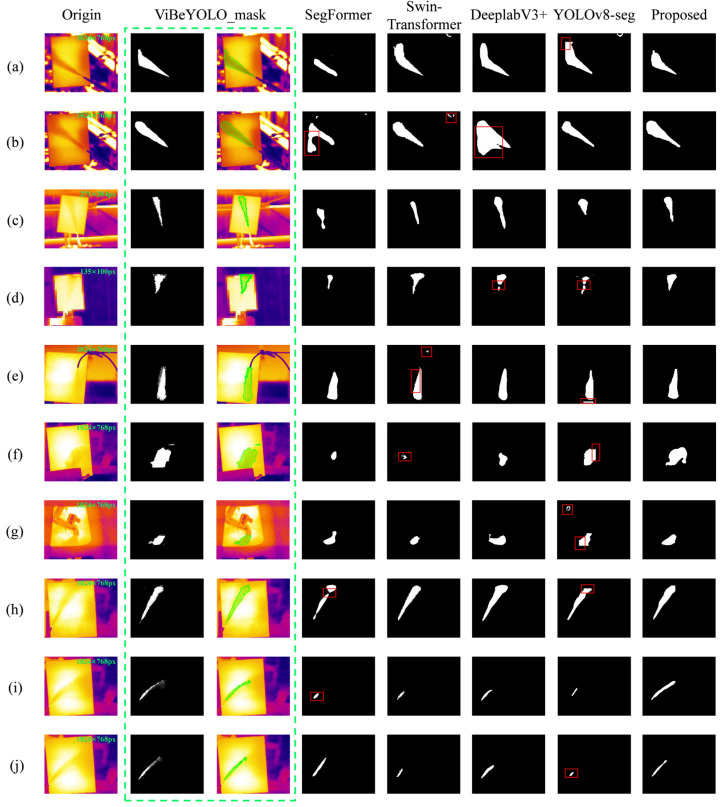
Visualization of network processing infrared image frame sequence. Experimental conditions (leakage orifice diameter ø, distance d, leakage scenario, wind speed v): (**a**) ø = 4 mm, d = 5 m; (**b**) ø = 1 mm, d = 5 m; (**c**) ø = 1 mm, d = 10 m; (**d**) ø = 1 mm, d = 15 m; (**e**) Thin hose leakage; (**f**) Occluded leakage orifice; (**g**) Leakage orifice in complex station structures; (**h**) ø = 1 mm, v = 0 m/s; (**i**) ø = 1 mm, v = 3 m/s; (**j**) ø = 1 mm, v = 5 m/s.

In this study, dynamic kernels and deformable convolutions were introduced into the segmentation head, enabling the generated masks to overcome the spatial limitations of anchor boxes and exhibit continuous and smooth changes at the edges. By combining ViBe and CRF-based confidence in the loss function, the model’s attention on the gas region’s boundary pixels is enhanced, reducing the roughness of transition regions and mitigating jaggedness in the mask boundaries. To visually verify the effectiveness of these optimizations, the segmentation results from different networks are displayed in the binary images shown in Figure 5.

In the experimental control groups of Figure 5, the gas internal pressure is unified at 0.5 MPa. Groups (a)–(d) represent the segmentation results for a leakage orifice diameter of 4 mm at a distance of 5 m, 1 mm at a distance of 5 m, 1 mm at a distance of 10 m, and 1 mm at a distance of 15 m, respectively. Groups (e)–(g) denote the leakage scenarios of a thin hose, an occluded leakage orifice, and a leakage orifice within complex structures of the station, respectively. Groups (h)–(j) represent the cases with a leakage orifice diameter of 1 mm under wind speeds of 0 m/s, 3 m/s, and 5 m/s, respectively. To provide a more intuitive quantitative validation of the generalization performance of the proposed algorithm across varying shooting distances and backgrounds, the pixel dimensions of each experimental image are annotated in green text at the top-right corner of the original images in Figure 5, serving as a visual reference. A similar approach is applied to the legends in subsequent figures.

From Figure 5, it can be seen that current mainstream semantic segmentation models can roughly localize the gas region, but they still have limitations in capturing the full features of the gas. In Figure 5, the red box highlights the regions where the comparison network demonstrates poor segmentation performance. The SegFormer and DeeplabV3+ models exhibit large-scale false detection in some experimental groups, leading to misclassification of background areas as foreground. The Swin-Transformer network tends to segment smaller areas as foreground, with noticeable jagged edges, indicating poor performance in handling boundary details. The original YOLOv8-seg model shows clear anchor box features in the segmented gas results, specifically in the form of excessive straight lines and right-angle shapes within the segmented region. This suggests that the network relies heavily on anchor boxes for mask generation, and this rigid constraint fundamentally conflicts with the diverse morphology of gas diffusion. In contrast, the improved model proposed in this study demonstrates the best segmentation accuracy and the lowest false detection rate in the processing of actual gas leakage images. It is more effective in capturing the variable characteristics of gas leakage, reflecting better adaptability to the gas diffusion morphology.

**Figure 6:**
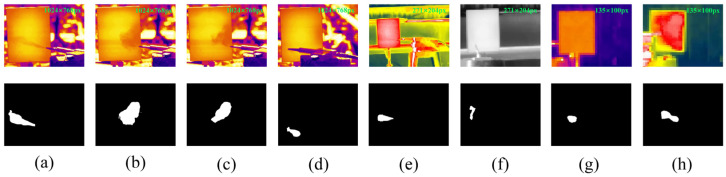
Segmentation results of the proposed model under low leakage rate. Experimental conditions (distance d, orifice diameter ø, wind speed v): (**a**) d = 5 m, ø = 1 mm, v = 1 m/s; (**b**) d = 5 m, ø = 1 mm, v = 5 m/s; (**c**) d = 5 m, ø = 2 mm, v = 1 m/s; (**d**) d = 5 m, ø = 2 mm, v = 5 m/s; (**e**) d = 10 m, ø = 1 mm, v = 1 m/s; (**f**) d = 10 m, ø = 1 mm, v = 5 m/s; (**g**) d = 15 m, ø = 1 mm, v = 1 m/s; (**h**) d = 15 m, ø = 1 mm, v = 5 m/s.

To further investigate the detection performance and accuracy of the proposed model, twelve groups of experiments were conducted focusing on the minimum leakage rate, with controlled variables including distance, orifice diameter, ambient wind speed, and minimum leakage volume. Partial experimental results are shown in Groups (a)–(h) of Figure 6. Specifically, Groups (a)–(d) correspond to scenarios with a 5 m distance, a 1 mm orifice diameter at wind speeds of 1 m/s and 5 m/s, and a 2 mm orifice diameter at wind speeds of 1 m/s and 5 m/s, respectively. Groups (e)–(f) represent cases with a 10 m distance and a 1 mm orifice diameter at 1 m/s and 5 m/s, while Groups (g)–(h) denote a 15 m distance and a 1 mm orifice diameter at 1 m/s and 5 m/s.

Notably, although the image resolution decreased with increasing distance, the trained segmentation network accurately segmented the gas regions in infrared images. At the maximum tested distance of 15 m, gas leakage was imperceptible to the naked eye in original images, and mask generation quality was suboptimal for some frames; thus, Groups (g)–(h) with superior mask quality were selected to validate model performance. The results demonstrate that the model’s segmentation outcomes matched the leakage orifice positions and exhibited similar shape and size to the frame-generated masks. While minor discrepancies existed at gas edges, the high segmentation accuracy was validated considering the minimal proportion of the intercepted leakage region in the entire image at this distance.

### 4.6. Discussion on Method Limitations

**Figure 7:**
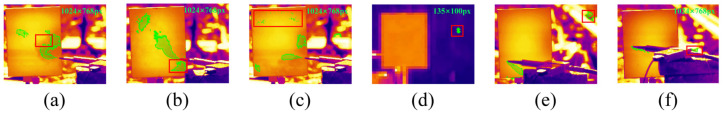
Illustration of mask generation inaccuracies.

To comprehensively explore the limitations of the adaptive ViBe and YOLOv8-det hybrid method in foreground segmentation and mask generation, this study evaluates its performance in terms of mask accuracy and computational efficiency. Figure 7 presents cases of mask generation errors on a custom dataset, with red boxes marking problematic regions.

In Figure 7a, partial gas regions fail to be accurately segmented as masks. This occurs because the continuous presence of gas in sequential frames activates the adaptive ViBe algorithm’s background update mechanism, causing temporary inaccuracies in foreground detection for specific frames. Figure 7b,c highlight false positives where sections of a constant-temperature plate are misclassified as gas regions. This error originates from transient thermal gradients generated by the plate’s annular heating module during prolonged operation. These gradients, combined with methane gas expansion’s heat-absorption effect, interfere with foreground detection. Moreover, the morphological similarity between thermal gradient diffusion and gas dispersion patterns leads to erroneous gas classification by the YOLOv8-det model.

Figure 7d–f depict scenarios where moving objects in the background introduce short-term small-area “ghosting” in some frames, leading to erroneous masks. While these factors constrain the method’s applicability to the custom dataset, the erroneous masks exhibit two critical attributes: spatially minimal coverage and extremely low occurrence rates. Considering the heightened complexity of gas flow patterns in intricate scenarios, the composite method’s performance does not necessarily imply a significant decline in mask generation quality under complex conditions. Post-training segmentation analysis confirms that such rare errors minimally affect model learning. Practically, isolated small-area gas segmentation artifacts are scarcely observed in final outputs, underscoring the method’s robustness despite these minor limitations.

The improved YOLOv8-seg segmentation network effectively eliminates non-physical artifacts in the original architecture, particularly addressing issues such as truncated segmentation regions induced by anchor box limitations. Nevertheless, during minimal gas leakage experiments, the proposed model occasionally failed to detect valid gas regions in certain frames. This limitation primarily arises from measurable degradation in infrared imaging quality under long-distance acquisition conditions with minimal leakage rates, compounded by insufficient representation of such extreme operational scenarios in the training dataset, which collectively lead to suboptimal segmentation outcomes.

To mitigate these constraints, future research requires supplementing the self-built dataset with samples of trace gas leakage in complex backgrounds, potential extreme leakage cases from oil and gas station environments, and leakage occurrences under diverse atmospheric conditions (such as high temperature/humidity and sandy weather). Concurrently, the segmentation model requires a balanced optimization strategy that strengthens the spatial coherence of gas recognition areas through noise suppression mechanisms while preserving sensitivity to subtle leakage signatures via multiscale feature enhancement.

## 5. Conclusions

This study proposes a low-cost annotation-based improved YOLOv8-seg network algorithm to address the challenges of large training annotation datasets for pixel-level segmentation networks and the anchor box constraint problem encountered by YOLOv8-seg when handling gas diffusion with diverse morphologies. First, the adaptive ViBe algorithm is integrated with YOLOv8-det to generate high-confidence training masks on unannotated infrared video sequences. A dynamic kernel segmentation head architecture is then designed, incorporating deformable convolutions and multi-head attention mechanisms to enhance the model’s ability to capture gas diffusion patterns. Finally, a ViBe-CRF confidence-weighted joint loss is introduced to replace the original loss function. The proposed model exhibits transferable applicability to all gases whose motion behavior can be captured by optical devices via specific band characteristics, demonstrating favorable cross-substance generalization ability. In future research, the integration of gas diffusion dynamics models with physics-based prior knowledge from visual perception can further refine the self-supervised segmentation network with physics augmentation for industrial detection applications. Based on the custom dataset, the conclusions of this study can be summarized as follows:

(1) A dynamic threshold-guided ViBe-YOLOv8 collaborative mask generation mech-anism. Experimental results demonstrate that using the dynamic threshold ViBe algorithm combined with the YOLOv8-det network achieves high accuracy in mask generation. This method transforms the substantial manual annotation workload into low-cost anchor box annotations, providing a novel weakly supervised solution for infrared gas detection.
(2) A dynamic confidence-weighted loss function is proposed, integrating masks gen-erated by the adaptive ViBe algorithm with CRF-optimized spatial consistency constraints. This approach effectively suppresses the interference of unreliable labels. Compared to the original loss function, the new loss function improves the DSC by 6.4% and the mIoU by 7.6%.
(3) A segmentation head structure is proposed that incorporates a dynamic kernel generation module, multi-core prediction branches, and an attention fusion module. This architecture enhances segmentation accuracy. The introduction of spatial offset fields and deformable convolutions addresses the anchor box-based segmentation problem in YOLOv8-seg when dealing with multi-morphology targets. The improved network structure leads to a 6.4% increase in the F1 score.

## Figures and Tables

**Table 1:** Main specifications of the infrared thermographic camera.

Performance	
Image frequency	30 Hz
Spectral range	3.2~3.5 µm
Thermal sensitivity	0.010 °C at 30 °C
Spatial resolution (IFOV)	0.65 mrad
Array format	1024 × 768

**Table 2:** Confusion matrix for gas leakage detection.

Predict Real	Gas Present	No Gas
Gas Present	TP	FP
No Gas	FN	TN

**Table 3:** Comparison of loss function performance.

Loss Function	F1/%	mIoU/%	Precision/%	Recall/%	GFLOPs	Latency/ms
BCE (Baseline)	67.4	59.8	73.1	62.5	42.2	5.9
BCE + DiceLoss	70.5	65.2	75.6	66.0	42.3	6.0
BCE + Dynamic Weighting	68.8	63.7	74.4	63.9	42.5	6.0
**Proposed**	**73.8 (6.4↑)**	**67.4 (7.6↑)**	**78.3 (+5.2↑)**	**69.7 (+7.2↑)**	**42.7**	**6.1**

The up arrow (↑) indicates an improvement achieved compared to the baseline algorithm.

**Table 4:** Comparison of segmentation results for different pixel-level segmentation models.

Arithmetic	F1/%	mIoU/%	Precision/%	Recall/%	GFLOPs	Latency/ms
YOLOv8-seg	67.4	59.8	73.1	62.5	42.2	19.5
SegFormer	64.8	58.7	67.1	62.7	27.2	91.5
Swin-Transformer	67.5	66.9	64.0	71.4	112.22	127.9
DeeplabV3+	69.4	62.6	66.1	73.1	37.9	32.9
**Proposed**	**73.8 (6.4** **↑)**	**67.4 (7.6** **↑)**	**78.3 (+5.2** **↑)**	**69.7 (+7.2** **↑)**	**42.7**	**20.1**

The up arrow (↑) indicates an improvement achieved compared to the baseline algorithm.

## Data Availability

Data will be made available upon request.
